# New York Academy of Medicine

**Published:** 1881-03-20

**Authors:** 


### *NEW YORK ACADEMY OF MEDICINE.*

At a meeting of the New York Academy of Medicine, held January 20, 1881, the following resolution was adopted:

*Resolved*, That a committee be appointed by the President to investigate the extent to which leprosy prevails in the United States.

The President appointed as such committee, Drs. H. G. Piffard, F. R. Sturgis, and G. H. Fox.

The committee are desirous of ascertaining the actual number of lepers in this country at the present time, and to that end respectfully request any physician who may know of the existence of a case in his neighborhood to communicate the fact to the chairman of the committee, at No. 10 West 35th Street, New York.

